# Inpatient treatment of GHB/GBL dependence: a case report

**DOI:** 10.1192/j.eurpsy.2023.1390

**Published:** 2023-07-19

**Authors:** M. Delic

**Affiliations:** Center for Treatment of Drug Addiction, University Psychiatric Clinic Ljubljana, Ljubljana, Slovenia

## Abstract

**Introduction:**

Gamma-hydroxybutyrate (GHB) and its precursor gamma-butyrolactone (GBL) are popular drugs of abuse used for their euphoric, (potential) anabolic, sedative, and amnestic properties. Daily use of GHB/GBL can lead to dependence and the possibility of a withdrawal syndrome on cessation which results in tremor, tachycardia, insomnia, anxiety, hypertension, delirium, and coma.

**Objectives:**

To describe the inpatient treatment and outcome of treatment of a patient with GHB/GBL dependence.

**Methods:**

A review of the case of patient reporting GHB/GBL dependence who was admitted for inpatient treatment.

**Results:**

The patient was using more potent substance GBL daily, 1.5 to 2 ml every two hours. She was using cannabis, alcohol, cocaine, and amphetamine-type stimulants additionally. Psychiatric comorbidities such as personality disorders, and eating disorders were recognized. Delirium developed after six hours of the last dose of GBL. The patient was treated with diazepam, clomethiazole, and atypical antipsychotics. She completed detoxification but stopped her treatment earlier. After two weeks she started to drink alcohol and after one month relapsed with GHB/GBL.

**Conclusions:**

GHB/GBL withdrawal can be severe and retention in the program is poor. Polysubstance use, psychiatric co-morbidities, and heavier GHB/GBL use as possible predictors of poor treatment outcomes need consideration in treatment planning.

**Disclosure of Interest:**

None Declared

